# Influences of club connectedness among young adults in Western Australian community-based sports clubs

**DOI:** 10.1186/s12889-020-08836-w

**Published:** 2020-05-19

**Authors:** Sharyn Burns, Melissa Evans, Jonine Jancey, Linda Portsmouth, Bruce Maycock

**Affiliations:** Collaboration for Evidence, Research and Impact in Public Health, School of Public Health, Curtin University, GPO Box U1987, Perth, Western Australia 6845 Australia

## Abstract

**Background:**

Along with physical benefits, community-based sport provides opportunities to enhance connectedness, an important protective factor of social and emotional health. However, young Australians participating in sport have been found to drink alcohol at higher levels than their non-sporting peers, and many clubs serve unhealthy food and beverages. This study explored the association between the dependent variable, level of alcohol consumption (AUDIT-C) and connectedness to club and other health behaviours among young people aged 18–30 years who play club sport in Western Australia.

**Methods:**

An online cross sectional survey measured levels of alcohol consumption (AUDIT-C), alcohol-related harm, connectedness (including volunteering and team cohesion), mental wellbeing, healthy food options and club sponsorship among young adults aged 18–30 years involved in sports clubs in Western Australia (*n* = 242). Relationships and association between the dependent variable (AUDIT-C) and independent variables were assessed.

**Results:**

Male sportspeople were more likely to drink alcohol at high-risk levels than females (*p* < .001), and respondents belonging to a club that received alcohol-related sponsorship were more likely to drink at high-risk levels (*p* = .019). Females were significantly more likely to want healthy food and beverage options provided at their clubs (*p* = 0.011). When all factors were considered team cohesion (*p* = 0.02), alcohol expectations (*p* = < .001), occurrences of experienced alcohol-related harm (*p* = <.001) and length of club membership (*p* = 0.18) were significant predictors of high-risk AUDIT-C (*R*^2^ = .34, adjusted *R*^2^ = .33, *F* (4, 156) = 20.43, *p* = <.001). High-risk AUDIT-C and club connectedness predicted strong team cohesion (*R*^2^ = .39, adjusted *R*^2^ = .39, *F* (2, 166) = 53.74, *p* = <.001).

**Conclusions:**

Findings from this study may inform policy and practice to enhance healthy behaviours among young adults participating in community sports clubs in Australia and other countries.

## Background

In many countries, community-based sport is an important part of the lives of many young people [1–3]. In Australia, there are over 50,000 sport and active recreation clubs, with those involved taking on the roles of player, coach, committee member, spectator or volunteer [4]. Sports involvement includes a range of individual and community-based benefits including connectedness [5].

Although benefits of sports club connectedness are discussed in some reports [6], few studies have measured associations between connectedness, alcohol consumption and other influences in the sports club setting. Connectedness provides a sense of belonging and it is believed having social ties to the community has links to positive outcomes such as positive mental health and health behaviour, less risk taking behaviour such as alcohol and other drug use, and better academic achievement for those in school [7–9]. Many club members play sport as well as volunteer with volunteers reporting higher levels of well-being, social connectedness, self-esteem and self-efficacy than non-volunteers [10].

However, people belonging to sporting clubs report higher levels of alcohol consumption compared to the general population [11]. In Australia [12, 13], and other countries, such as the US [14] and New Zealand [15], strong social and cultural norms are associated with alcohol consumption and sport.

Social alcohol consumption has been associated with team cohesion and bonding in team sports [16] with association between team social cohesion and risky alcohol consumption correlated to the frequency of team social events involving alcohol [17]. Despite these associations, players reporting positive team cohesion are more likely to continue participation [18]. There is also evidence to suggest involvement in sporting clubs provides young people protection from the uptake of risky drinking [19] due to the reduced levels of ‘unsupervised leisure time’ and boredom, resulting in less time and inclination for anti-social behaviour [20]. The social cohesion and support found in community sporting clubs may also act as a protective factor [12].

Club ethos and environment are important factors in attracting and retaining members. Clubs often aim to attract families making sports clubs an important community venue and social centre for all ages [12]. For many community-based sports clubs, the bar provides not only access to alcohol and revenue for the club, but a community hub, which fosters connectedness and socialisation [13, 21]. However, the balance between alcohol access and responsible drinking is important. A study of 33 Australian community football clubs found having the bar open for more than 4 hours; serving intoxicated patrons; and alcohol promotions, to be associated with lower levels of perceived club safety and participation [13]. Provision of food contributes to club engagement and is a strategy of responsible alcohol consumption [11, 12], however, many sports clubs [22] and sporting events provide energy-dense and nutrient poor food and beverages [23] which are often supported by industry sponsorship [24]. Despite this there have been significant successes in reduction of risky alcohol consumption and related harm among Australian sports clubs when environmental and policy changes are implemented (*n* = 43 intervention; *n* = 45 control community football clubs) [11].

Sports clubs play an important role in the socio-cultural environment of many countries [23], with complex associations between club connectedness and health behaviours. There is little doubt as to the physical and mental health benefits of sports participation [25] or of the benefits of connectedness to mental and social wellbeing [7]. However, despite some success with structured interventions [11] the culture of risky alcohol consumption and community sports is of concern [26]. This study explored the association between the dependent variable, level of alcohol consumption (AUDIT-C) and connectedness to club and other health behaviours among young people aged 18–30 years who play club sport in Western Australia. This paper extends the literature around alcohol consumption and sports club participation to consider the influence of club connectedness.

## Method

A cross sectional online survey including questions related to alcohol consumption, alcohol-related harm, healthy eating options, sponsorship, club connectedness (including volunteering) and mental wellbeing was administered. Ethics approval was received from the Curtin University Human Research Ethics Committee (HRE2016–0241). Interested participants clicked the survey link and were provided an online Participant Information Form describing the project. Participants were asked to indicate consent via an online question before they were able to proceed to the survey. Helplines and support services were provided for participants.

### Participants

Young adults aged between 18 and 30 who live in Western Australia and participate in team sports were invited to participate in the online survey, which was open for a 2-month period. Recruitment occurred through convenience sampling using a dedicated project Facebook Page, club Facebook Pages and newsletters, other social media sites and snowball technique. Due to the convenience and snowball sampling methods employed, it is unknown how many clubs posted the survey information. A new Facebook business page was created for the study to enhance communication and recruitment, a strategy successfully employed in another alcohol study with young people [27]. Paid Facebook advertising assisted recruitment. An optional prize draw was offered as an incentive for participation. Efforts to ensure that duplicate survey completion was detected included investigation of similar or identical email addresses and IP addresses.

### Instrumentation

#### Demographics

Demographic questions included age, gender (male, female, other), postcode, ethnicity, employment and student status. Age and location were restricted to ensure that only the intended target group completed the survey.

#### Involvement in sport

Involvement in sport was determined by four questions that identified the main sport participants played during the last 12 months, the length of involvement and weekly time commitment.

#### Availability of alcohol

To determine availability of alcohol, participants were asked if their club had alcoholic drinks available for purchase, and how often the bar was open. Participants were also asked if food is served with alcohol. These questions were developed for this survey.

#### Alcohol consumption

The Alcohol Use Disorders Identification Test-Consumption (AUDIT-C) was employed to measure levels of alcohol consumption [28]. AUDIT and AUDIT-C are well validated and widely used tools developed for the World Health Organisation [28–30]. The AUDIT-C includes three consumption questions, is considered a sensitive indicator of alcohol consumption [31] and has been used with young sports people [32] and student samples [33]. Consistent with other research with young adults scores were collapsed into binary categories: low-risk (< 6) and high-risk (≥6) consumption [34].

#### Alcohol-related harm

Experienced, witnessed and second-hand alcohol-related harms were measured using previously validated scales [35, 36]. These questions were modified slightly to reflect consumption associated with teammates or sports club members during the last season. Harms as a result of other club members’ alcohol consumption were measured using a 10-item scale (5-point Likert scale) [36, 37]. Witnessed harms included an eight-item scale on a 5-point Likert scale [35]. Experienced harms, harms associated with personal alcohol consumption, were measured using a validated version of the adapted Alcohol Problems Scale [36]. The 16-item scale included a 3-point Likert scale. Alcohol expectancies were measured using the validated Brief Comprehensive Effects of Alcohol Scale (B-CEOA), a nine-item scale with responses agree, neither agree or disagree or disagree (score range 9–27) [38].

#### Volunteering

Involvement in volunteering was determined by questions of time committed, years of service and future planned volunteering. Volunteer motivation was measured via a validated scale (20 items; 7-point Likert) [39].

#### Connectedness and cohesion

Club connectedness was determined using a five-item Likert scale [40] previously used to measure connectedness [41]. Perceptions of fitting in with team and club members were measured using a validated four-item Likert scale [42].

Team cohesion was measured using an adapted version of the Group Environment Questionnaire (GEQ) (18 items) focusing on individual attraction and group integration [43]. The GEQ was analysed as a whole scale (team cohesion) and by the previously validated four constructs: Individual attractions to the Group-Task (an individual’s feelings about personal involvement with the group task); Individual attractions to the Group-Social (individual’s feelings about personal group social interactions); Group Integration Task (individual’s perceptions around group unification of tasks); and Group Integration-Social (individual’s perception of the group socially) [43]. The GEQ has previously been used to assess social connectedness and team cohesion in young people from sports clubs [44–46].

#### Mental wellbeing

Mental wellbeing was measured using the Warwick-Edinburgh Mental Well-being Scale (WEMWBS) [47]. The 14 item scale has been used to measure the mental wellbeing of young adults who play sport [48, 49] and has demonstrated sound validity with a student sample (Cronbach alpha 0.89) [47].

#### Food and drink availability

To measure availability of food and beverages at their sports club, participants were asked if their club had a bar, canteen or vending machine and availability and frequency of use. Perception of the healthiness of the food and beverage options, and what they would like provided, was asked. Questions were adapted from an Australian survey targeting community sports clubs [50]. The following was included to clarify examples of food choices: “Examples of healthy food and beverages include fruit, salad rolls/sandwiches, unsalted nuts and water. Examples of unhealthy food and beverages soft drinks, energy drinks, pies, sausage rolls, hotdogs, hot chips, lollies, chocolate bars and crisps”.

#### Sponsorship

Adapted questions were used to explore the presence and impact of sponsorship in community sporting clubs questions [51, 52]. These questions had previously been used to measure alcohol-sponsorship [51] and modified to measure non-alcohol sponsorship among Australian sportspeople [52]. Questions focused on sponsorship from alcohol-related or food and beverage industries and the type of sponsorship received. Participants were asked if their club/organisation/team received any sponsorship. Those who recalled sponsorship were provided a list of sponsors (for example, liquor store, brewery, hotel etc.) and then a question asking what type of sponsorship was received (for example, cash, uniforms, alcohol etc.). Separate questions were asked for alcohol and non-alcohol-related sponsorship.

### Data analysis

Prior to conducting analyses total scores were calculated for scales and sub-scales. Nominated sports included Australian Rules Football League (AFL), hockey, netball, soccer, Ultimate Frisbee and Other. The ‘other’ group consisted of sports that had a low number of respondents and included American football, basketball, bouldering, cheerleading, cricket, floorball, gymnastics, judo, polocrosse, Quidditch, rowing, Rugby League, Rugby Union, softball, street roller hockey, swimming, taekwondo, tennis, volleyball and water polo.

The cross-sectional design of the survey allowed for the examination of relationships between variables [53]. Univariate analysis was used to determine the association between the dependent variable (AUDIT-C) and independent variables (sport, gender, age, location, student status, length of club membership, volunteer roles, alcohol-related sponsorship, non-alcohol-related sponsorship, experienced harm, second-hand harm, witnessed harm, club connectedness, team cohesion and mental wellbeing). Variables were compared between two levels of alcohol consumption (low- and high-risk drinkers) and gender (male and female). Chi square and Mann Whitney U and ANOVA tests were used to test relationships; Bonferroni correction was applied; Spearman’s correlation measured association between variables. Dancey and Reidy’s [54] interpretations guided the strength of correlations. Using these guidelines a correlation coefficient of + 1 and − 1 was considered perfect; + 0.9 - + 0.7 and − 0.9 - -0.7 as strong; + 0.6 - + 0.4 and – 0.6 - -0.4 as moderate; + 0.3 − + 0.1 and − 0.3 - -0.1 as weak; and 0 as no correlation. Significance was determined at *p* < 0.001 and moderate significance at *p* < 0.05.

Multiple regression analysis explored associations between the dependent variable (AUDIT-C) and independent variables (see above). Due to data being non-normally distributed non-parametric statistics were used to conduct the analyses. When analysing the data by gender, ‘other’, was excluded due to a low number of respondents for this option. Data were analysed using SPSS (version 25).

## Results

Respondents (*n* = 242) were most likely to play Australian Football League (AFL), netball, hockey, soccer and Ultimate Frisbee; and to be female (approx. 60%), with an average age of 26 (*SD* = 6.9). Almost half (46%) of respondents studied at university full-time, and a third (32%) had been a member of their club for more than 5 years. Not all respondents completed all questions (see tables).

Fifty-four per cent of respondents (*n* = 114/209) volunteered at their club, with 34% volunteering 2–3 times a week or more. Overall, female respondents (57%) volunteered more than males (50%); however, males volunteered on a more frequent basis than females. Forty-six per cent of males volunteered 2–3 times a week, compared to 27% of females. The majority of respondents (64%) reported average or above average mental wellbeing scores. Eighty-two percent of respondents reported a strong connection to their clubs, and 45% of respondents reported high team cohesion.

Overall, 60% of respondents’ clubs had a bar and a canteen (*n* = 106/177), 16.9% (*n* = 30) a canteen only, and 3.4% (*n* = 6) a bar only, with the canteen (100%) and bar (87%) most likely to be open on match days. For clubs with a bar most served meals (69%) or snacks (16.8%). Clubs received sponsorship from alcohol-related industry (26%) and from other businesses (57.5%) and food outlets (19%).

A third (31%) of respondents indicated they purchased food and beverages from their canteen once a week. Sixty seven percent of respondents indicated their club did not have a vending machine. Only 7% of respondents’ clubs sold mostly healthy food and beverage options, and 62% said that they would like healthier options available.

### Alcohol consumption

An AUDIT-C score was computed for 171 participants. Of these 32% (*n* = 55) reported high-risk levels of alcohol consumption. Males were more likely to report high-risk levels of consumption compared to females (49% vs. 21% respectively). Nearly two-thirds (64%) of respondents (*n* = 168) had experienced one form of alcohol-related harm whilst drinking alcohol with their teammates or other sports club members, for example, having a hangover (56%), feeling sick or vomiting (38%), memory loss (20%), and driving a car while under the influence of alcohol (12%). At least one incident of alcohol-related harm had been witnessed by 40% of respondents and 43% had experienced second-hand harm as a result of teammates’ drinking (Table 1).

**Table 1:** Demographics and influencing factors for low and high-risk alcohol consumption

	*Low-risk* *N (%)*	*High-risk* *N (%)*	*Total* *N (%)*	*P value*
Sport				*p* = .020*
AFL	19 (16.4)	21 (38.2)	40 (23.4)	
Hockey	21 (18.1)	4 (7.3)	25 (14.6)	
Netball	22 (19)	9 (16.4)	31 (18.1)	
Soccer	13 (11.2)	4 (7.3)	17 (9.9)	
Ultimate Frisbee	12 (10.3)	2 (3.6)	14 (8.2)	
Other***	29 (25)	15 (27.3)	44 (25.7)	
Total	116	55	171	
Gender				*p* = .001*
Male	34 (29.3)	33 (60.0)	67 (39.2)	
Female	81 (69.8)	22 (40.0)	103 (60.2)	
Other	1 (0.9)	0	1 (.06)	
Total	116	55	171	
Age				*p* = .966
18–24 years	67 (57.8)	31 (57.4)	98 (57.6)	
25–30 years	49 (42.2)	23 (42.6)	72 (42.4)	
Total	116	54	170	
Location				*p* = .329
Metro	110 (94.8)	50 (90.9)	160 (93.6)	
Regional/rural	6 (5.2)	5 (9.1)	11 (6.4)	
Total	116	55	171	
Student status				*p* = .014*
Non-student	44 (37.9)	28 (50.9)	72 (42.1)	
University – full-time	59 (50.9)	21 (38.2)	80 (46.8)	
University – part-time	12 (10.3)	1 (1.8)	13 (7.6)	
TAFE – full-time	0 (0)	1 (1.8)	1 (0.6)	
TAFE – part-time	0 (0)	2 (3.6)	2 (1.2)	
Other	1 (0.9)	2 (3.6)	3 (1.8)	
Total	116	55	171	
Length of club membership				*p* = .303
Less than 1 year	26 (22.4)	6 (10.9)	32 (18.7)	
1–2 years	30 (25.9)	15 (27.3)	45 (26.3)	
3–5 years	28 (24.1)	14 (25.5)	42 (24.6)	
More than 5 years	32 (27.6)	20 (36.4)	52 (30.4)	
Total	116	55	171	
Volunteer roles				*p* = .437
Yes	58 (50.0)	31 (56.4)	89 (52.0)	
No	58 (50.0)	24 (43.6)	82 (48.0)	
Total	116	55	171	
Alcohol-related sponsorship				*p* = .019*
Yes	22 (19.5)	21 (38.9)	43 (25.7)	
No	68 (60.2)	22 (40.7)	90 (53.9)	
Unsure	23 (20.4)	11 (20.4)	34 (20.4)	
Total	113	54	167	
Non-alcohol related sponsorship				*p* = .229
Yes	60 (53.1)	36 (66.7)	96 (57.5)	
No	21 (18.6)	6 (11.1)	27 (16.2)	
Unsure	32 (28.3)	12 (22.2)	44 (26.3)	
Total	113	54	167	
Experienced harm	M 1.9	M 4.0	M 2.6	*p* < .001**
	SD 3.5	SD 3.7	SD 3.7	
	CI 1.3–2.6	CI 2.9–5.0	CI 2.1–3.2	
Second-hand harm	M 1.5	M 1.39	M 1.5	*p* = .199
	SD 3.5	SD 1.9	SD 3.1	
	CI 0.8–2.2	CI 0.8–1.9	CI 1.0–1.9	
Witnessed harm	M 1.9	M 2.3	M 2.1	*p* = .403
	SD 4.4	SD 3.8	SD 4.2	
	CI 1.2–2.8	CI 1.2–3.3	CI 1.4–2.7	
Alcohol expectations	M 20.7	M 22.9	M 21.4	*p* = .005*
	SD 4.6	SD 3.4	SD 4.4	
	CI 19.8–21.6	CI 21.9–23.8	CI 20.7–22.1	
Club connectedness	M 21.4	M 21.8	M 21.5	*p* = .143
	SD 3.1	SD 3.4	SD 3.1	
	CI 20.8–21.9	CI 20.9–22.8	CI 21.1–21.9	
Team cohesion (GEQ)	M 117.7	M 124.0	M 118.6	*p* = .043*
	SD 21.8	SD 24.8	SD 23.1	
	CI 113.5–121.9	CI 117.3–130.7	CI 115.3–121.9	
Individual attractions to the Group-Social (ATG-S)	M 32.6	M 35.3	M 33.5	*p* = 0.034*
	SD 7.4	SD 8.6	SD 7.9	
	CI 31.2–33.9	CI 32.9–37.7	CI 32.3–34.7	
Individual attractions to the Group-Task (ATG-T)	M 29.4	M 29.8	M 29.6	*p* = 0.722
	SD 5.32	SD 6.9	SD 5.9	
	CI 28.5–30.4	CI 27.7–31.6	CI 28.7–30.4	
Group Integration Task (GI-T)	M 33.1	M 34.1	M 33.4	*p* = 0.416
	SD 7.3	SD 7.0	SD 7.2	
	CI 31.7–34.4	CI 32.2–35.9	CI 32.3–34.5	
Group Integration-Social (GI-S)	M 22.4	M 24.9	M 23.2	*p* = 0.14
	SD 6.1	SD 6.4	SD 6.3	
	CI 21.2–23.5	CI 23.1–26.6	CI 22.2–24.11	
Mental Wellbeing	M 52.7	M 53.1	M 52.3	*p* = .782
	SD 8.5	SD 7.5	SD 8.7	
	CI 51.2–54.3	CI 50.9–55.1	CI 51.1–53.6	

*. *p* < 0.05 level

**. *p* < 0.01 level

***Other = American football, basketball, bouldering, cheerleading, cricket, floorball, gymnastics, judo, polocrosse, Quidditch, rowing, Rugby League, Rugby Union, softball, street roller hockey, swimming, taekwondo, tennis, volleyball and water polo

Low and high-risk AUDIT-C scores were compared with key demographic variables. High-risk drinking was proportionally more likely to be reported by AFL players (38.2%) (*p* = .020); males (60.0%) (*p* = .001); respondents who were not students (50.9%) (*p* = .014) and those belonging to a club that received alcohol-related sponsorship (38.9%) (*p* = .019). High-risk drinkers were significantly more likely to experience alcohol-related harm (M 4.0; SD 3.7) (*p* < .001); have higher alcohol expectations (M 22.9; SD 3.4) (*p* = .005); and report higher team cohesion (M 124.0; SD 24.8) (*p* = .043). There was no significant difference between low and high-risk drinking and age, location, length of club membership, volunteer roles, non-alcohol related sponsorship, second-hand harm, witnessed harm, club connectedness and mental wellbeing.

### Gender differences

Males (45.7%) were significantly more likely to play AFL while females were more likely to play netball (32%) and hockey (20.0%) (*p* < .001). While there was no significant difference between uptake of volunteer roles and gender, male respondents were significantly more likely to volunteer on a more frequent basis (*p* = .037). Female respondents (68.4%) were moderately significantly more likely to want more healthy food and beverage options at their club (53.4%) (*p* = .011). Male respondents were significantly more likely to report higher AUDIT-C scores (M 5.0; SD 2.3) (*p* = .001), experience alcohol related-harm (M 3.2; SD 3.4) (*p* = .003), second-hand alcohol-related harm (M 2.1; SD 3.9) (*p* = .006), and witness alcohol-related (M 3.2; SD 5.3) (*p* = .004). There was no significant difference between gender and length of club membership, alcohol expectations, club connectedness, team cohesion and mental wellbeing (Table 2).

**Table 2:** Demographics and influencing factors and gender *(male and female)*

	*Male* *N (%)*	*Female* *N (%)*	*Total* *N (%)*	*P value*
Sport				*p* < .001**
AFL	37 (45.7)	10 (8.0)	47 (22.8)	
Hockey	3 (3.7)	25 (20.0)	28 (13.6)	
Netball	0 (0)	40 (32.0)	40 (19.4)	
Soccer	12 (14.8)	11 (8.8)	23 (11.2)	
Ultimate Frisbee	4 (4.9)	12 (9.6)	15 (7.8)	
Other***	25 (30.9)	27 (21.6)	52 (25.2)	
Total	81	125	206	
AUDIT-C category				*p* < .001**
Low-risk	34 (50.7)	81 (78.6)	115 (67.6)	
High-risk	33 (49.3)	22 (21.4)	55 (32.4)	
Total	67	103	170	
Length of club membership				*p* = .761
Less than 1 year	14 (17.3)	24 (19.0)	38 (18.4)	
1–2 years	22 (27.2)	33 (26.2)	55 (26.6)	
3–5 years	22 (27.2)	27 (21.4)	49 (23.7)	
More than 5 years	23 (28.4)	42 (33.3)	65 (31.4)	
Total	81	126	207	
Volunteer roles				*p* = .357
Yes	41 (50.6)	72 (57.1)	113 (54.6)	
No	40 (49.4)	54 (42.9)	94 (45.4)	
Total	81	126	207	
Time spent volunteering				*p* = .037*
2–3 times a week or more	18 (46.2)	19 (26.8)	37 (33.6)	
Once a week	13 (33.3)	21 (29.6)	34 (30.9)	
Once a month or less	8 (20.5)	31 (43.7)	39 (35.5)	
Total	39	71	110	
Healthy food options				*p* = .011*
Yes, absolutely	10 (16.7)	34 (35.8)	44 (28.4)	
Yes	22 (36.7)	31 (32.6)	53 (34.2)	
No	24 (40.0)	19 (20.0)	43 (27.7)	
Unsure	4 (6.7)	11 (11.6)	15 (9.7)	
Total	60	95	155	
AUDIT-C score	M 5.0	M 3.9	M 4.5	*p* = .001*
	SD 2.3	SD 3.2	SD 2.3	
	CI 4.4–5.6	CI 3.5–4.3	CI 4.0–4.7	
Experienced harm	M 3.2	M 2.3	M 2.6	*p* = .003*
	SD 3.5	SD 3.8	SD 3.7	
	CI 2.4–4.1	CI 1.5–3.0	CI 2.1–3.2	
Second-hand harm	M 2.3	M 1.1	M 1.5	*p* = .006*
	SD 3.9	SD 2.4	SD 3.1	
	CI 1.2–3.1	CI 1.1–1.5	CI 1.1–1.9	
Witnessed harm	M 3.2	M 1.4	M 2.1	*p* = .004*
	SD 5.3	SD 3.2	SD 4.2	
	CI 1.9–4.5	CI .7–1.9	CI 1.4–2.7	
Alcohol expectations	M 22.1	M 21.1	M 21.5	*p* = .313
	SD 3.7	SD 4.7	SD 4.4	
	CI 21.2–23.0	CI 20.1–21.9	CI 20.8–22.1	
Club connectedness	M 21.7	M 21.4	M 21.5	*p* = .455
	SD 3.3	SD 3.0	SD 3.1	
	CI 20.9–22.5	CI 20.8–21.9	CI 21.1–21.9	
Team cohesion (GEQ)	M 119.2	M 118.2	M 118.6	*p* = .608
	SD 25.6	SD 21.6	SD 23.2	
	CI 113.2–125.3	CI 114.3–122.2	CI 115.3–121.9	
Individual attractions to the Group-Social (ATG-S)	M 34.3	M 32.4	M 33.1	*p* = 0.308
	SD 9.1	SD 7.6	SD 7.6	
	CI 32.1–36.4	CI 30.9–33.8	CI 31.9–34.3	
Individual attractions to the Group-Task (ATG-T)	M 29.0	M 29.1	M 29.1	*p* = 0.823
	SD 6.9	SD 5.9	SD 6.3	
	CI 287.4–30.6	CI 28.0–30.2	CI 28.2–40.0	
Group Integration Task (GI-T)	M 32.3	M 33.8	M 33.2	*p* = 0.305
	SD 7.6	SD 6.9	SD 7.2	
	CI 30.5–34.0	CI 32.6–35.1	CI 32.2–34.3	
Group Integration-Social (GI-S)	M 23.7	M 22.9	M 23.2	*p* = 0.14
	SD 6.73	SD 5.9	SD 6.2	
	CI 22.13–25.29	CI 21.9–24.1	CI 22.3–24.1	
Mental Wellbeing	M 54.2	M 51.3	M 52.4	*p* = .073
	SD 8.0	SD 9.0	SD 8.8	
	CI 52.3–56.1	CI 49.6–53.0	CI 51.1–53.7	

*. *p* < 0.05 level

**. *p* < 0.01 level

***Other = American football, basketball, bouldering, cheerleading, cricket, floorball, gymnastics, judo, polocrosse, Quidditch, rowing, Rugby League, Rugby Union, softball, street roller hockey, swimming, taekwondo, tennis, volleyball and waterpolo

### Multiple regression

When all factors were considered, team cohesion (*p* = 0.02), alcohol expectations (*p* = < .001), occurrences of experienced alcohol-related harm (*p* = <.001) and length of club membership (*p* = 0.18) accounted for a significant 34.4% of the variability in AUDIT-C scores (*R*^2^ = .34, adjusted *R*^2^ = .33, *F* (4, 156) = 20.43, *p* = <.001). In combination, club connectedness (*p* < .001) and AUDIT-C (*p* = .145) score accounted for a significant 39.3% of the variability in team cohesion (*R*^2^ = .39, adjusted *R*^2^ = .39, *F* (2, 166) = 53.74, *p* = <.001).

### Correlations between variables

AUDIT-C score was moderately significantly positively correlated with club connectedness (*r*_*s*_ = .18, *p* < .05, two-tailed, *N* = 171) and team cohesion (*r*_*s*_ = .22, *p* < .01, two-tailed, *N* = 171), however these correlations were weak. When team cohesion was collapsed to measure individual and group task and social constructs, moderately significant, but weak, positive correlations were found between AUDIT-C and Group Integrated-Social (*r*_*s*_ = .22, *p* < .01, two-tailed, *N* = 171); Individual Attractions Group Social (*r*_*s*_ = .25, *p* < .01, two-tailed, *N* = 171); and Individual Attractions Group Task (*r*_*s*_ = .16, *p* < .01, two-tailed, *N* = 171). Moderate positive relationships were reported for AUDIT-C score with experienced harm (*r*_*s*_ = .50, *p* < .001, two-tailed, *N* = 168), alcohol expectations (*r*_*s*_ = .38, *p* < .001, two-tailed, *N* = 167) and drinking alcohol with teammates (*r*_*s*_ = .44, *p* < .001, two-tailed, *N* = 160) (Table 3).

**Table 3:** Correlations between variables

	AUDIT-C score	Club connectedness	Experienced harm	Second-hand harm	Witnessed harm	Wellbeing	Team cohesion	GI-T	GI_S	ATG-S	ATG-T	Alcohol expectations	Club membership	Volunteer roles	Drinking with teammates
AUDIT-C score	___														
Club connectedness	.18*	___													
Experienced harm	.50**	.13	___												
Second-hand harm	.10	−.15	.32**	___											
Witnessed harm	.12	−.16*	.42**	.65**	___										
Wellbeing	.04	.28**	.10	.01	.12	___									
Team cohesion	.22**	.54**	.22**	−.15	−.13	.29**	___								
GI-T	.117	.54**	.08	.17*	.23**	.18**	.85**	___							
GI_S	.22**	.43**	.58**	.08	.02	.16**	.74**	.522*	___	.					
ATG-S	.25**	.49**	.24**	−.025	−.034	.27**	.84**	.55**	.55**	___					
ATG-T	.16**	.48**	.15*	−.29**	−.21	.27**	.83**	.74**	.42**	.61**	___				
Alcohol expectations	.38**	.01	.23**	.16*	.23**	.09	.02	.09	.02	.-.08	.14*	___			
Club membership	.12	.05	.03	.06	.03	.03	.04	.09	−.06	.07	.02	−.01	___		
Volunteer roles	−.05	−.14	−.19*	−.13	−.12	−.06	−.14	−.11	−.15*	−.16*	−.14	.001	−.30**	___	
Drinking with teammates	.44**	.20*	.45**	.20*	.33**	.06	.29**	.13	.34**	.31**	.25**	.22**	.10	−.20**	___

*p < 0.05

**p < 0.001

Interpretation of Spearman’s Correlation Coefficients (54) 
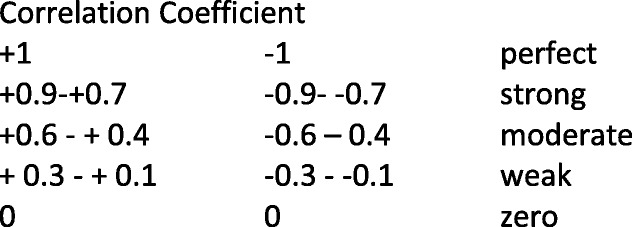

Club connectedness and team cohesion were moderately correlated (*r*_*s*_ = .54, *p* < .001, two-tailed, *N* = 190). Mental wellbeing score was significantly positively correlated with team cohesion (*r*_*s*_ = .29, *p* < .001, two-tailed, *N* = 179) and club connectedness (*r*_*s*_ = .28, *p* < .001, two-tailed, *N* = 179), however correlations were weak. All four constructs of team cohesion were also positively correlated with club connectedness and mental wellbeing.

All alcohol-related harms were significantly positively correlated, with most demonstrating moderate correlations. Experienced harm was significantly positively correlated with second-hand harm (*r*_*s*_ = .32, *p* < .001, two-tailed, *N* = 168) and witnessed harm (*r*_*s*_ = .42, *p* < .001, two-tailed, *N* = 168), and second-hand harm and witnessed harm were significantly positively correlated (*r*_*s*_ = .65, *p* < .001, two-tailed, *N* = 168). Drinking alcohol with teammates was significantly positively correlated with experienced alcohol-related harm (*r*_*s*_ = .45, *p* < .001, two-tailed, *N* = 157), second-hand harm (*r*_*s*_ = .20, *p* < .05, two-tailed, *N* = 157) and witnessed harm (*r*_*s*_ = .33, *p* < .001, two-tailed, *N* = 157) (Table 3).

## Discussion

Associations between club connectedness, alcohol consumption and other behaviours among young adults involved in community sports clubs were explored. Similar to other studies of sportspeople in Australia [52], New Zealand [55] and Ireland [56] males in this study reported to drink alcohol at riskier levels than females. These studies used the 10-item AUDIT as opposed to AUDIT-C to measure levels of alcohol consumption. Young Australian adult males are reported to consume alcohol at higher levels than females of the same age, as demonstrated within public [1] and university populations [35].

Strong team cohesion was a predictor of high-risk alcohol consumption. Team cohesion has been identified as a significant predictor of alcohol consumption amongst university sportspeople in the UK (*n* = 1785) [16], while teammates influence and the team environment has been found to influence higher levels of alcohol consumption amongst sports players [15, 49, 57]. However a study amongst female US college athletes (*n* = 174) found no significant association between team social cohesion (computed using an average of the Individual Attraction to Group-Social and Group Integration-Social scores) and hazardous drinking [17]. When team cohesion in this study was analysed using the four constructs; social, as opposed to task oriented constructs accounted for higher correlations to alcohol consumption, however although moderately significant these correlations were weak.

Club connectedness and AUDIT-C scores were also found to significantly correlate to team cohesion, particularly the social team cohesion constructs. To our knowledge, no studies have reported on the association between club connectedness and team cohesion. This is worthy of further exploration.

Respondent mental wellbeing was positively, although weakly, correlated with team cohesion and club connectedness, supporting previous findings [16, 49, 58]. The social nature of participating in team sport is believed to contribute to improved health and mental wellbeing [59], and research amongst US college students has revealed that connectedness acts as a protective factor against depression for athletes, which may be the result of having a social network and team support [60].

Volunteers are considered the most important resource for community sports clubs and provide opportunity to enhance club connectedness [61]. Approximately 65% of participants volunteered once a week or more. This is higher than the national findings of 39% of adult Australians, and only 8.4 and 10.5% of young adults aged 18–24 and 25–34 years respectively, volunteering at least once a week with their club [62]. In contrast to other studies [62–64], a higher percentage of females volunteered for their club than males. However, these studies explored volunteering in the general public [64] or explored sport volunteer rates across the life span and did not specifically examine gender by age groups [62, 63]. A negative correlation between the uptake of volunteer roles and length of club membership was found. Similarly, others have found similar associations, suggesting volunteers may give up volunteer roles if dissatisfied or perceive that they have fulfilled their obligations [65] .

Females in this study were significantly more likely to want more healthy food options available at their club than males. While the promotion of food and beverages has been explored in some studies [66, 67], and another has explored stakeholders views [23]; there is limited research exploring sportspeople’s attitudes towards the availability of healthy food in sports clubs. However, Australian female athletes are more opposed to unhealthy food advertising in sport than male athletes [68] and females have a stronger interest in healthy eating than males [69].

Participants were more likely to indicate that their team or club received non-alcohol related sponsorship (57.5%) compared to alcohol-related sponsorship (25.7%). It is unknown if recall of sponsorship is accurate, however another Australian study using similar measures found 31% (*n* = 204) of sportspeople to report some sponsorship, with most (95%; *n* = 194) receiving alcohol-related sponsorship. In contrast to the current study fewer (*n* = 26; 13%) received non-alcohol related sponsorship and *n* = 16 (8%) received both [52]. Respondents belonging to a club that received alcohol-related sponsorship were significantly more likely to consume alcohol at hazardous levels, supporting previous findings from Australia [52], New Zealand [51] and the UK [70] and in a systematic review [71]. Food and beverage sponsors, especially for energy-dense, nutrient-poor foods high-energy, do target sports venues and clubs [23]. More research to explore the impacts among young adults would be beneficial.

### Recommendations for intervention

The findings of this study highlight the importance of sports clubs as community-based environments, which have the potential to enhance positive physical, social and emotional health outcomes [72, 73]. Some sports clubs have implemented strategies to create environments that recognise and capitalise on their role as community hubs [21]. The Good Sports program has demonstrated some positive changes to alcohol consumption especially among AFL and cricket clubs in Australia when policy-related strategies were implemented [11, 74]. After a 4 month intervention including behavioural and policy-related strategies an Irish controlled trial of sports clubs in two RCT found no reduction in alcohol consumption, or AUDIT scores, although there was reduction in the number of alcohol related harms reported [56].

A qualitative study (*n* = 22) found alcohol to be seen to help strengthen team cohesion by providing an opportunity for teammates to socialise and bond [75]. This supports other findings of association between levels of alcohol consumption and team social cohesion [17] and the culture of alcohol consumption and sports club involvement in many countries [12, 13]. These findings, along with those of this study highlight the importance of clubs to provide opportunity for teammates to socialise together in order to strengthen team cohesion, in environments that support responsible alcohol consumption.

Despite the majority of respondents indicating that they would like more healthy food and beverage options available in their clubs, only a small number of clubs in this study sold mostly healthy options. Club representatives of community football clubs in Australia are in favour of providing a greater range of healthy food options in their club [24]. Increasing the availability and promoting healthy food and beverages in community football clubs has been found to increase sales of these products [67]. Intervention could involve encouraging young adults to eat healthier to complement their athletic and sporting goals. Such an intervention would need to address potential barriers including time restraints, financial implications and peer influences [76]. Future interventions need to address potential barriers for selling healthy foods in sports canteens, which include cost, perceived lack of demand, perishability of healthy foods, storage requirements and lack of knowledge of canteen staff [24].

The significant association between alcohol-related sponsorship and alcohol consumption in sport players provides further evidence to support replacement alcohol sponsorship in sports. There have been calls for government regulation of alcohol sponsorship in sport in Australia, with community members supporting the replacement of alcohol sponsorship in community sport clubs if compensated for lost revenue [50, 77]. Banning of tobacco sponsorship in sport provides examples of effective strategies that could be applied to alcohol sponsorship [78].

Policy development can assist sports clubs in implementing health promotion initiatives; however, it is important for clubs to receive support from health agencies, which can include training or advice and sample policies [72]. Capacity building [79] and organisational change [80] are also important factors in helping clubs adopt and implement health promotion strategies.

The findings of this study highlight the associations between club connectedness, team cohesion and mental wellness. Young adults were active volunteers, further enhancing their connection to club. Participants wanted healthy food and beverage options available at their clubs. However, connectedness and team cohesion were associated with higher levels of alcohol consumption and related harms. Given the role of alcohol in Australian sporting culture [11] this is not surprising. Health promotion intervention should harness the enthusiasm of young adults involved with their sports clubs. Based on the findings of this study key strategies for healthier clubs include:

- Provide acceptable alternatives to high levels of alcohol consumption to enhance team cohesiveness and club connectedness;
- Replace alcohol sponsorship (ensuring clubs do not lose revenue);
- Capitalise on young adults’ desire for healthier food and beverage options; and
- Support and promote volunteer opportunities within clubs.

## Limitations

Females were more likely to respond to the survey, however, other studies of similar age groups have also reported a majority of female respondents [35, 42, 81]. The small sample is a limitation of this study. Recent data suggests 68% of Australian 18–24 year olds participated in sport-related activity in the last 12 months [82]. Budget and time constraints influenced recruitment. Recruitment relied on self-selection, which may bias findings. The data collection was conducted during the winter months with the majority of respondents playing winter sports, and the majority from the Perth metropolitan area. Consequently, the sample may not be representative of the community sporting population aged 18–30 years within Western Australia. A higher proportion of male participants, and greater representation from rural sports clubs would strengthen the study. Future research could involve conducting the survey during the summer months, which would allow for a comparison by seasons, promoting the survey in rural and regional areas of Western Australia and including more structured recruitment procedures.

## Conclusion

Previous studies have highlighted the association between high levels of alcohol consumption and participation in community sports clubs; however, there has been little focus on the association between connectedness, alcohol consumption and other health enhancing behaviours such as availability of healthy food and beverage options. The findings of this study highlight the benefits of club connectedness in creating a community and providing a sense of belonging and connection. The association between club connectedness, team cohesion, in particular team social cohesion, and higher levels of alcohol consumption, highlights the need for tailored interventions to focus on enhancing responsible alcohol consumption while maintaining club connectedness and team cohesion. The study findings also highlight the importance of attracting and retaining volunteers, especially young adults. The opportunity for sports clubs to promote other health enhancing behaviours, particularly associated with food and beverage consumption was also evident. It is unrealistic to expect clubs to be able to navigate the changes required and implement comprehensive health promotion programs without both structural and educative support.

## Data Availability

The datasets used and/or analysed during the current study are available from the first author on reasonable request.

## Abbreviations

AUDIT-C: Alcohol Use Disorders Identification Test-Consumption
B-CEOA: Brief Comprehensive Effects of Alcohol Scale
WEMWBS: Warwick-Edinburgh Mental Well-being Scale
GEQ: Group Environment Questionnaire
AFL: Australian Football League
